# Preclinical Studies of Mesenchymal Stem Cell (MSC) Administration in Chronic Obstructive Pulmonary Disease (COPD): A Systematic Review and Meta-Analysis

**DOI:** 10.1371/journal.pone.0157099

**Published:** 2016-06-09

**Authors:** Xiangde Liu, Qiuhong Fang, Huijung Kim

**Affiliations:** 1 Pulmonary, Critical Care, Sleep and Allergy Medicine, Department of Internal Medicine, University of Nebraska Medical Center, Omaha, Nebraska, United States of America; 2 Department of Pulmonary and Critical Care, Beijing Chaoyang Hospital, The Capital Medical University, Beijing, China; 3 Pulmonary and Critical Care Division, WonKwang University, Sanbon Medical Center, Seoul, Korea; University Hospital of Salamanca, SPAIN

## Abstract

**Background:**

In the last two decades, mesenchymal stem cells (MSCs) have been pre-clinically utilized in the treatment of a variety of kinds of diseases including chronic obstructive pulmonary disease (COPD). The aim of the current study was to systematically review and conduct a meta-analysis on the published pre-clinical studies of MSC administration in the treatment of COPD in animal models.

**Methods and Results:**

A systematic search of electronic databases was performed. Statistical analysis was performed using the Comprehensive Meta-Analysis software (Version 3). The pooled Hedges’s g with 95% confidence intervals (95% CIs) was adopted to assess the effect size. Random effect model was used due to the heterogeneity between the studies. A total of 20 eligible studies were included in the current systematic review. The overall meta-analysis showed that MSC administration was significantly in favor of attenuating acute lung injury (Hedges’s g = -2.325 ± 0.145 with 95% CI: -2.609 ~ -2.040, *P* < 0.001 for mean linear intercept, MLI; Hedges’s g = -3.488 ± 0.504 with 95% CI: -4.476 ~ -2.501, *P* < 0.001 for TUNEL staining), stimulating lung tissue repair (Hedges’s g = 3.249 ± 0.586 with 95% CI: 2.103~ 4.394, *P* < 0.001) and improving lung function (Hedges’s g = 2.053 ± 0.408 with 95% CI: 1.253 ~ 2.854, *P*< 0.001). The mechanism of MSC therapy in COPD is through ameliorating airway inflammation (Hedges’s g = -2.956 ± 0.371 with 95% CI: -3.683 ~ -2.229, *P*< 0.001) and stimulating cytokine synthesis that involves lung tissue repair (Hedges’s g = 3.103 ± 0.734 with 95% CI: 1.664 ~ 4.541, *P*< 0.001).

**Conclusion:**

This systematic review and meta-analysis suggest a promising role for MSCs in COPD treatment. Although the COPD models may not truly mimic COPD patients, these pre-clinical studies demonstrate that MSC hold promise in the treatment of chronic lung diseases including COPD. The mechanisms of MSCs role in preclinical COPD treatment may be associated with attenuating airway inflammation as well as stimulating lung tissue repair.

## Introduction

Over the last two decades, tremendous progress has been made in the field of regenerative medicine and stem cell biology [1]. Mesenchymal stem cells (MSCs) are multi-potent stem cells that have fibroblast-like morphology and the capacity to differentiate into chondrocytes, osteoblasts, adipocytes and muscle cells under different micro-environmental conditions, culture media, and supplements [2, 3]. In addition to their regenerative properties, MSCs have recently been shown to have unique immune-modulatory and anti-inflammatory properties that render the MSCs as potential treatment options for a variety of kinds of inflammatory disorders including chronic obstructive pulmonary disease (COPD).

COPD is the third-leading cause of death in the United States [4, 5]. Despite recent advances in the treatment of symptoms with new pharmaceutical drugs and molecules, there remains no effective treatment to attenuate disease progression or reverse the COPD and emphysematous changes. Over the past decade, MSCs isolated from various tissues including bone marrow, adipose, or cord blood, have been shown to lack immunogenicity and thus, can be used for allogeneic or autologous cellular treatment in a variety of diseases. In this context, studies have demonstrated that MSCs have anti-inflammatory and immune-modulatory effects in diverse types of tissue injury and allergic inflammation [6, 7]. MSCs are now known to have potent beneficial effects in animal models of many types of lung injury including cigarette smoke-induced or elastase-induced COPD/emphysema [8–10], bleomycine-induced fibrosis [11, 12], bronchopulmonary dysplasia [13, 14], ventilator-induced lung injury [15], and bacterial pneumonia [16, 17]. Much of these preclinical data support the therapeutic potential of MSCs in the animal models of human diseases including COPD.

Based on the findings of preclinical studies on MSC administration in COPD animal models, a multicenter double-blind placebo-controlled Phase II trial of allogeneic MSC infusions for patients with moderate to severe COPD (FEV_1_/FVC < 0.70, 30% < FEV_1_ < 70%) have recently been completed by Weiss et al [18]. This trial was based on the hypothesis that the immune-modulating actions of MSCs would decrease pulmonary, and perhaps systemic, inflammation associated with COPD, thus improving lung function, dyspnea and quality of life. However, the result of this clinical trial was disappointing and found a lack of even a trend for efficacy of MSC administration in COPD despite significantly reduced serum C-reaction protein (C-RP) levels in the patients who received MSC administration [18].

Therefore, the current study was designed to systematically review pre-clinical studies of MSC administration in the experimental models of COPD and to examine the pooled effect of MSCs in reducing tissue damage and stimulating tissue repair in the animal models of COPD.

## Materials and Methods

### Data sources

This systematic review and meta-analysis followed the Preferred Reporting Items for Systematic Reviews and Meta-analyses (PRISMA) criteria [19]. Relevant literature was searched for with the following phrases: “mesenchymal stem cell(s)” and “COPD”, “mesenchymal stem cell(s)” and “emphysema”, “mesenchymal stromal cell(s)” and “COPD”, or “mesenchymal stromal cell(s)” and “emphysema” in the sites of PubMed, Embase and Web of Science. The search was limited to English. Relevant studies were also identified by hand-searching the references of included articles. Literature search was performed by the following authors: Xiangde Liu and Qiuhong Fang

### Inclusion and exclusion criteria

Studies were included in the current systematic review and meta-analysis if: 1) Studies examined the relationship between MSCs and COPD or emphysema in animal models, 2) Studies contained full text articles.

Studies were excluded if: 1) Insufficient publications existed to perform a systematic review and meta-analysis, 2) A second publication of similar studies in a different journal from the same research group, 3) *Ex vivo* or *in vitro* studies were conducted, 4) Studies utilizing MSC conditioned medium (MSC-CM), 5) Studies lacking measurement data and thus meta-analysis was not able to be performed.

### Data extraction

All three authors (XL, QF, HK) were involved in data extraction. Information and data were carefully extracted from all included literature according to the inclusion and exclusion criteria as aforementioned. Data include first author name, publication date, country, source of MSCs, recipient animal species, total number of cases or replication of the experiment, study design and parameters observed.

### Statistical analysis

The following forms of data were used for the data entry: 1) Mean, standard deviation (SD), number of animals in control group and number of animals in MSC administration group, 2) Sample size of control or MSC administration group and *P* value of comparison between the two groups. The strength of MSC effect on COPD or emphysema lung tissue repair or other biological effects was measured by Hedges’s g. A random effect model was applied due to the significant heterogeneity of the data collected. The heterogeneity between studies was assessed by the Q-test and I^2^ statistics, and *P* < 0.10 and I^2^ > 50% was considered as heterogeneous between the studies [20]. All meta-analysis was performed using the Comprehensive Meta-analysis software (Version 3, NJ, USA).

## Results

### Study features

**Fig 1:**
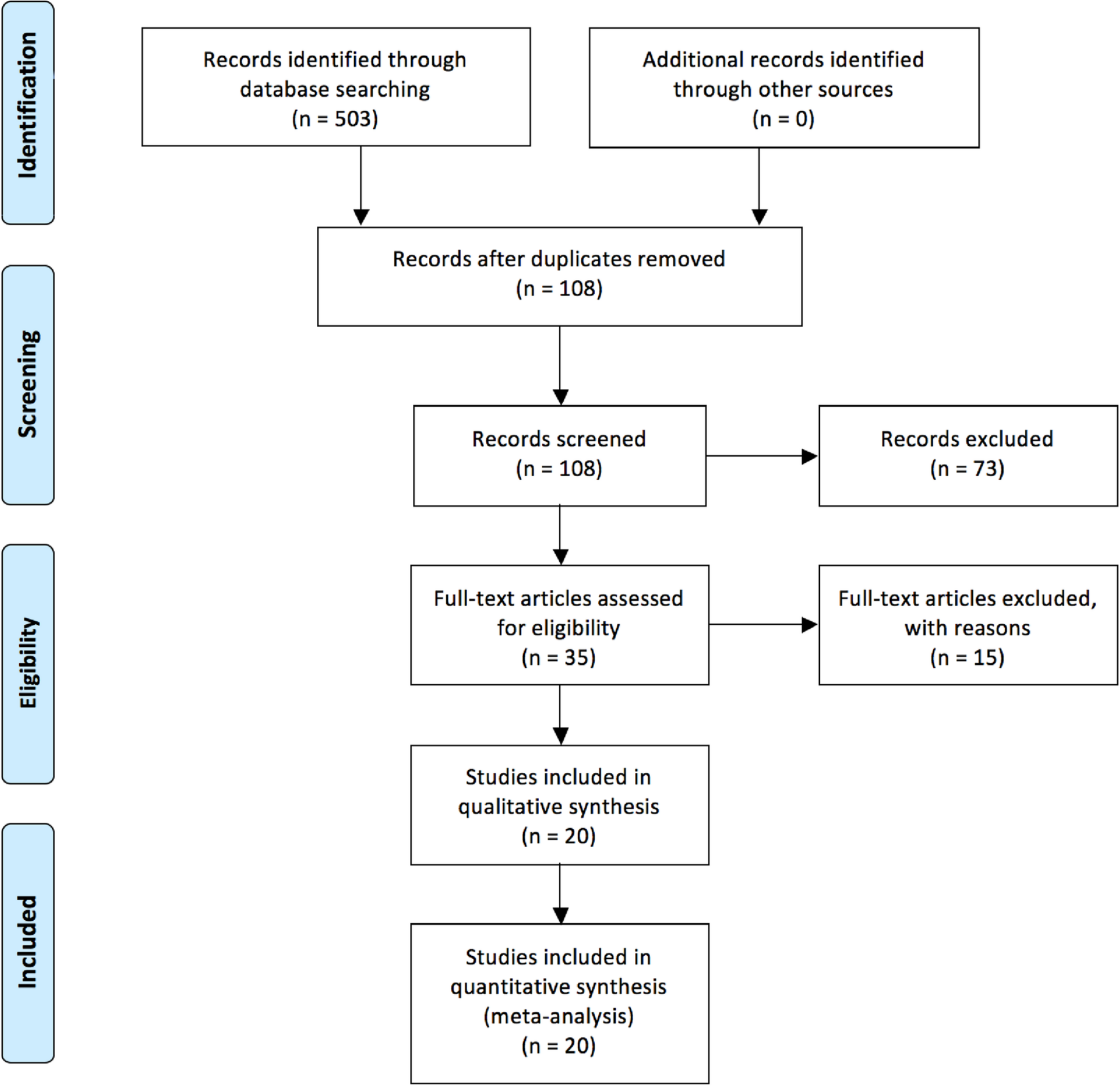
Flow diagram of literature search and eligible publication selection.

The process of selecting literature is outlined in **Fig 1**. After careful review of the abstracts of publications, a total of 36 full-text articles were retrieved. The full-text articles were independently assessed by all three authors. Twenty one articles were included in the systematic review and meta-analysis, as shown in **Table 1**, including studies of human bone marrow MSC (BM-MSC, n = 1), human adipose stromal cells (ASC, n = 1), human cord blood derived MSC (n = 1) or human tubal MSC (n = 1) [21–24], rat BM-MSC (n = 7) [9, 25–30], rabbit bone marrow derived mesenchymal stem cells (n = 1) [31], rat adipose derived stromal cells (n = 2) [32, 33], guinea pig adipose derived MSCs (n = 2) [34, 35], rat amniotic fluid derived MSC (n = 1) [36], and mice BM-MSC or adipose-derived MSC or lung tissue MSC (n = 4) [8, 10, 37, 38]. Among the 21 articles 8 are from China, 4 are from Japan, 2 are from Korea, 2 are from Brazil, 2 are from Iran, one is from Canada, one from the USA, and one is from Taiwan.

**Table 1:** Characteristics of included twenty one papers.

First author	Country	Year	MSC source	Recipients	COPD	Delivery	MSC dose & time	Parameters evaluated
Shigemura N	Japan	2006	Rat ASC	Rat	PPE	IV	5x10^7^/0.2mL	TUNEL, PCNA, RAC index
							1 wk after PPE	Factor VII, HGF, PaO2
								Maximum running,
Yuhgetsu H	Japan	2006	Rabbit	Rabbit	PPE	IB	10^8^/2mL	TUNEL, alveolar space
			BM-MSC				24 h after PPE	Ki-67 positivity
								BALF total cell, macrophage
Zhen GH	China	2008	Rat	Female rat	Papain	IV	4x10^6^/0.4mL	MLI, TUNEL
			BM-MSC				Simultaneously	
Zhen GH	China	2010	Rat	Female rat	Papain	IV	4x10^6^/0.4mL	MLI, TUNEL, Caspase-3
			BM-MSC				2 h after papain	VEGF-A
Huh JW	Korea	2011	Rat	Rat	CS	IV	6x10^5^/0.3mL	MLI, TUNEL
			BM-MSC				6 m after CS	
Katsha AM	Japan	2011	C57Blk6	C57Blk6	PPE	IT	5x10^5^/0.2mL	Lm, destructive index
			BM-MSC				14 d after PPE	IL-1β, IL-1β mRNA
								HGF mRNA, EGF mRNA
Schweitzer KS	USA	2011	Human	Rat	CS	IV	3x10^5^ cells	Lung macrophage, PMN
			ASC				2 m after CS	Caspase3, lung volume
								alveolar surface area
Furuya N	Japan	2012	Rat ASC	Rat	PPE	IV	2.5x10^6^/0.5mL	Lm, PaO2, HGF, CINC-1
							7 d after PPE	IL-1β,
Guan XJ	China	2013	Rat	Rat	CS	IT	6x10^6^/0.15mL	MLI, TUNEL, Caspase3
			BM-MSC				7 wk after CS	Vital capacity, FEV100
								MMP-9, MMP-12,
								TGF-β1, VEGF,
Antunes MA	Brazil	2014	C57Blk6	C57Blk6	PPE	IT and IV	1x10^5^ cells	Normal lung volume (%)
			BM-MSC				3 wk after PPE	Hyperinflation (%)
			AD-MSC					Lm, TUNEL
			L-MSC					Neutrophil
Feizpour A	Iran	2014	Guinea pigs	Guinea pigs	CS	IT and IV	10^6^/0.3mL	EC50 methacholine
			ASC				Day 1 and 14	Serum or BALF IL-8
								Blood or BALF WBC
Ghorbani A	Iran	2014	Guinea pigs	Guinea pigs	CS	IT and IV	10^6^/0.3mL	Emphysema score
			ASC				Day 1 and 14	BALF thiol, serum MDA
								Blood neutrophil, lymph
Li X	China	2014	Human	Rat	CS	IV	3x10^6^ cells	Lm, Trichrome
			iPS-MSC				Day 29 and 43	
			BM-MSC					
Li YQ	China	2014	Rat	Rat	CS + LPS	IT	4x10^5^/0.2mL	MLI, TUNEL
			AFD-MSC				4th and 8th wk	Mean alveolar area
Tibboel J	Canada	2014	C57Blk6	C57Blk6	PPE	IT	5x10^5^/0.2mL	MLI, Dynamic compliance
			BM-MSC			IV	1x10^5^/0.1mL	Mean Forced Expiratory
							Before & after PPE	Flow
Zhang WG	China	2014	Rat	Rat	CS	IV	4x10^6^/0.2mL	MLI, TUNEL, IL-6
			BM-MSC				Day 20 and 62	
Zhao YM	China	2014	Rat	Rat	CS + LPS	IV	5x10^6^ cells	Mean alveoli number
			BM-MSC				Day 36	Pulmonary alveolar area
Chen YB	Taiwan	2015	C57Blk6	C57Blk6	PPE	IV	?	MLI, VEGF mRNA, HSP70
			BM-MSC				14 d after PPE	Whole body plethysmograph
Gu W	China	2015	Rat	Rat	CS	IT	6x10^6^/0.15mL	MLI, COX-2 mRNA, PGE2
			BM-MSC				7 wk after CS	Inflammation score,
								IL-6, IL-10
Kim YS	Korea	2015	Human	C57Blk6	PPE	IV	Various dose	MLI, VEGF
			CBD-MSC				7 d after PPE	
Peron JP	Brazil	2015	Human	C57Blk6	CS +	IP or	1x10^6^ cells	BALF total cell, neutrophil
			Tubal MSC		Irradiation	Intranasal	Day 60 and 67	Airway mucus, collagen

ASC: adipose tissue derived stromal/stem cell; BM-MSC: bone marrow-derived mesenchymal stem cell

AD-MSC: adipose derived mesenchymal stem cell; L-MSC: lung tissue derived mesenchymal stem cell

CBD-MSC: cord blood derived mesenchymal stem cell; CS: cigarette smoke; PPE: porcine pancreatic elastase

IB: intra-bronchial; IV: intravenous; IT: intra-tracheal; IP: intra-peritoneal

### Results of overall systematic review

The first study of rodent MSC administration in a COPD model was published by Shigemura et al from Japan in 2006 [25, 32]. However, most studies on COPD therapy with MSC administration were published in 2014 (n = 8). Overall, administration of MSCs demonstrated that MSCs have therapeutic benefit in both structural and functional outcomes in the COPD animal models, which were prepared either by elastase instillation or cigarette smoke exposure. Sources of MSCs were from human, rabbit, rat, guinea pigs or mouse and delivered to the recipients either through intravenous (IV) injection, intra-tracheal (IT) or intra-bronchial (IB) instillation, intra-peritoneal injection or intranasal instillation. One study compared the efficiency of different MSC sources and delivery routes [8]. The authors found that IT administration of BM-MSC was superior to IV injection in terms of reducing alveolar hyperinflation or collagen fiber content in the lung. They also found that IV administration of lung tissue derived MSCs resulted in immediate death of the recipient mice while IV administration of BM-MSC or adipose derived MSC did not [8]. Most recently, Kim et al reported that a minimum number of 5x10^4^ MSCs was required to achieve therapeutic effect [23].

### Effect of MSC administration on lung injury and repair in COPD animal models

**Fig 2:**
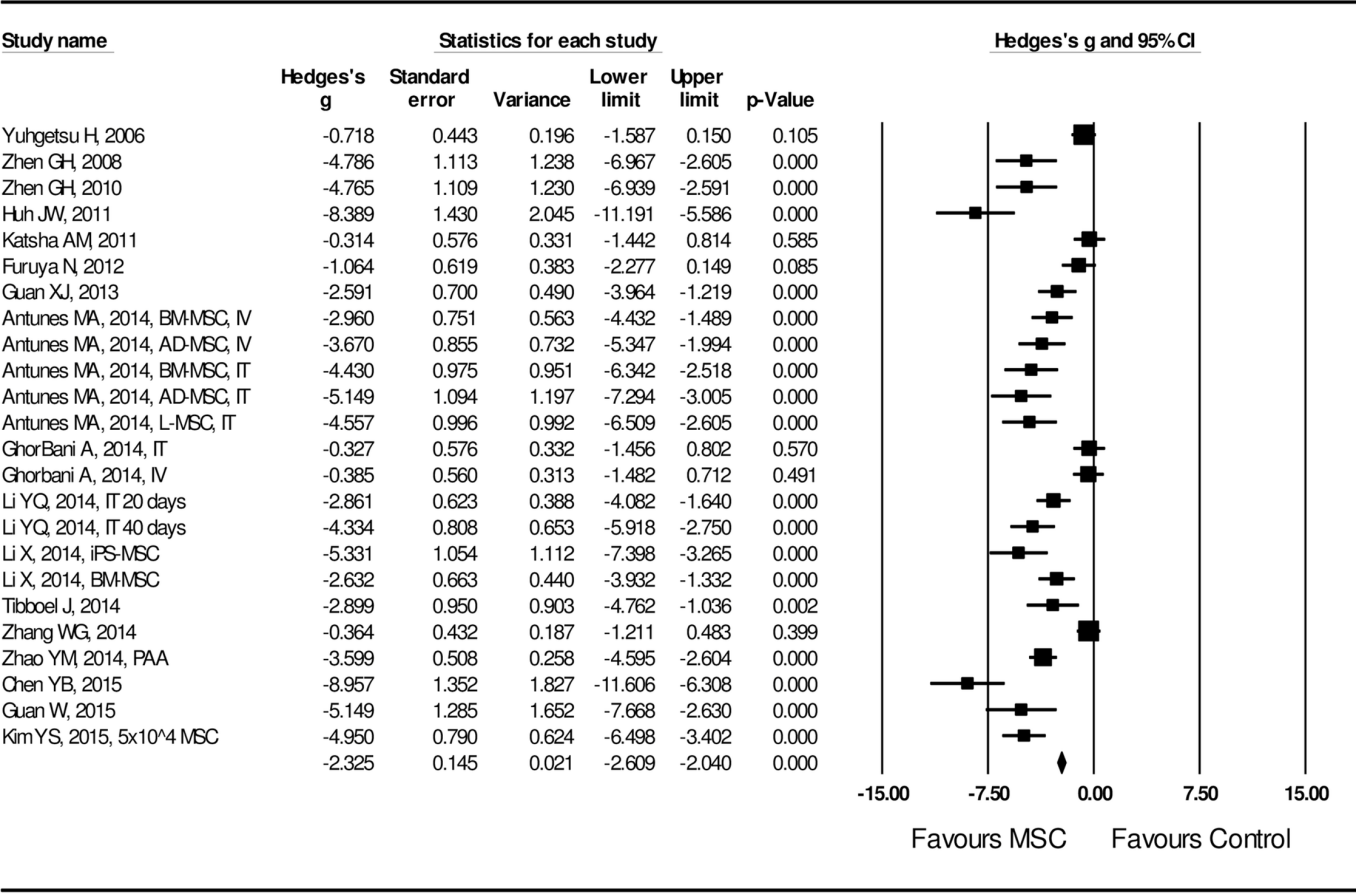
Forest plot for the MSC effect on mean linear interception (MLI). A random effect model was used due to significant heterogeneity of publications (I^2^ = 87.5, *P* < 0.01). Effect size was assessed by Hedges’s g and 95% CI, and the effect on MLI reduction was in favor of MSC treatment (Hedges’s g = -2.325 ± 0.145, 95% CI: -2.609~-2.040, *P* < 0.001) compared to control, which was the COPD model without MSC treatment.

**Fig 3:**
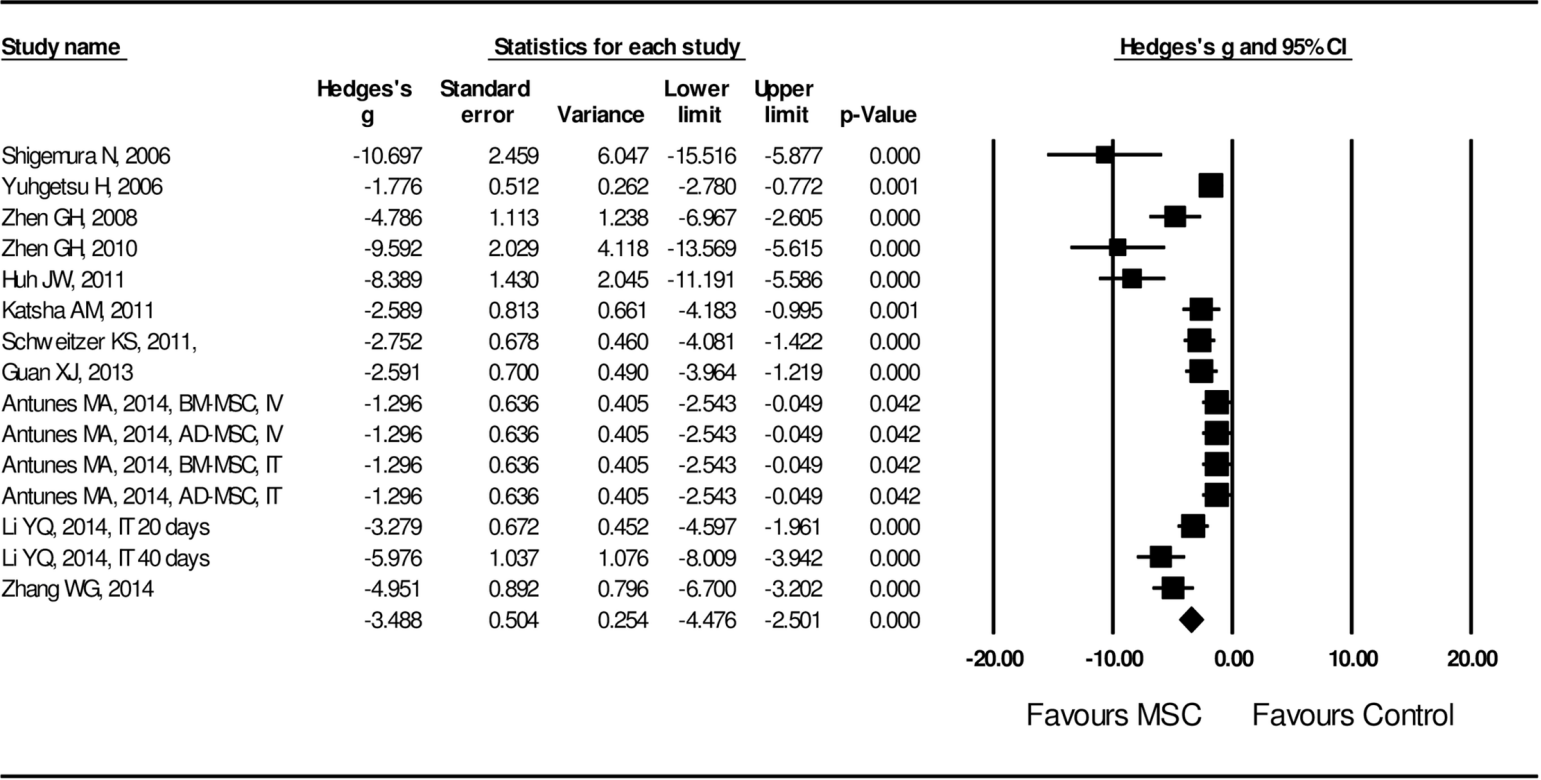
Forest plot for the effect of MSCs on TUNEL positivity. A random effect model was used due to significant heterogeneity of publications (I^2^ = 82.7, *P* < 0.01). Effect size was assessed by Hedges’s g and 95% CI, and the inhibitory effect on TUNEL positivity was in favor of MSC treatment (Hedges’s g = -3.488 ± 0.504, 95% CI: -4.478~-2.501, *P* < 0.001) compared to control group, which was the COPD model without MSC treatment.

**Fig 4:**
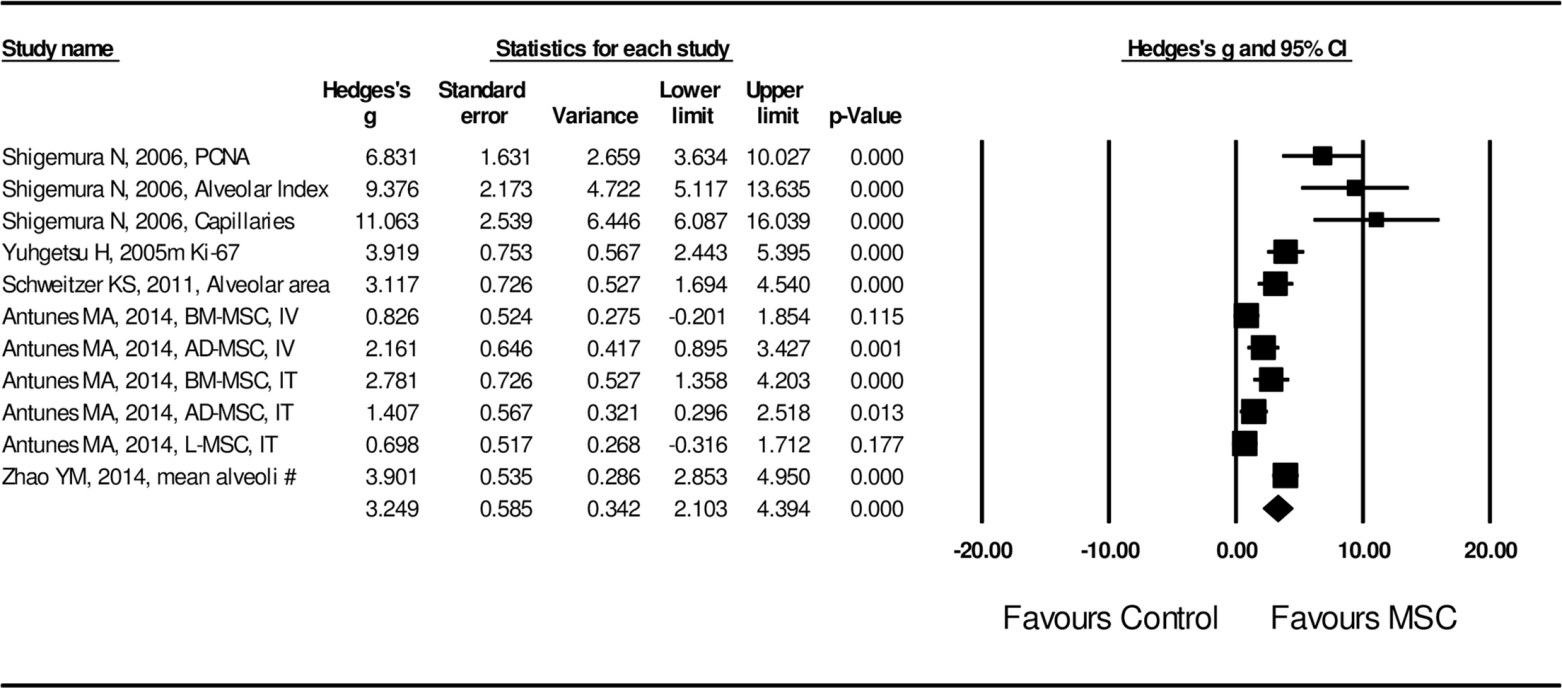
Forest plot for the effect of MSCs on lung tissue repair parameters. A random effect model was used due to significant heterogeneity of publications was observed (I^2^ = 83.2, *P* <0.01). Effect size was assessed by Hedges’s g and 95% CI, and the stimulatory effect on lung tissue repair was in favor of MSC administration (Hedges’s g = 3.249 ± 0.586, 95% CI: 2.103~4.394, *P* < 0.001). Control group was the COPD model without MSC treatment.

Effect size of MSC administration on lung structural injury was examined by length of mean linear interception (MLI) and positivity of TUNEL staining in the lung tissue. A random effect model was adopted in assessing the effect size of MSCs on MLI length and TUNEL positivity due to the high heterogeneity of the studies (I^2^ = 87.5 for MLI length and I^2^ = 82.7 for TUNEL positivity, *P* < 0.01). Effect size of MSC transplantation on MLI was significantly (Hedges’s g = -2.325 ± 0.145 with 95% CI: -2.609 ~ -2.040, *P* < 0.001, **Fig 2**) in favor of MSC treatment. Effect size of MSC administration on TUNEL positivity was also significant (Hedges’s g = -3.488 ± 0.504 with 95% CI: -4.478 ~ -2.501, *P* < 0.001, **Fig 3**) by random model of assessment in favor of MSC treatment. Effect size of MSC on lung tissue repair was evaluated by PCNA positivity, Ki-67 positivity, radial alveolar count index, factor VII for capillary assessment, alveolar surface area, and percent of normal lung. As shown in **Fig 4**, effect of MSC administration on lung tissue repair was significantly (Hedges’s g = 3.249 ± 0.586 with 95% CI: 2.103 ~ 4.394, *P* < 0.001) in favor of MSC treatment.

**Fig 5:**
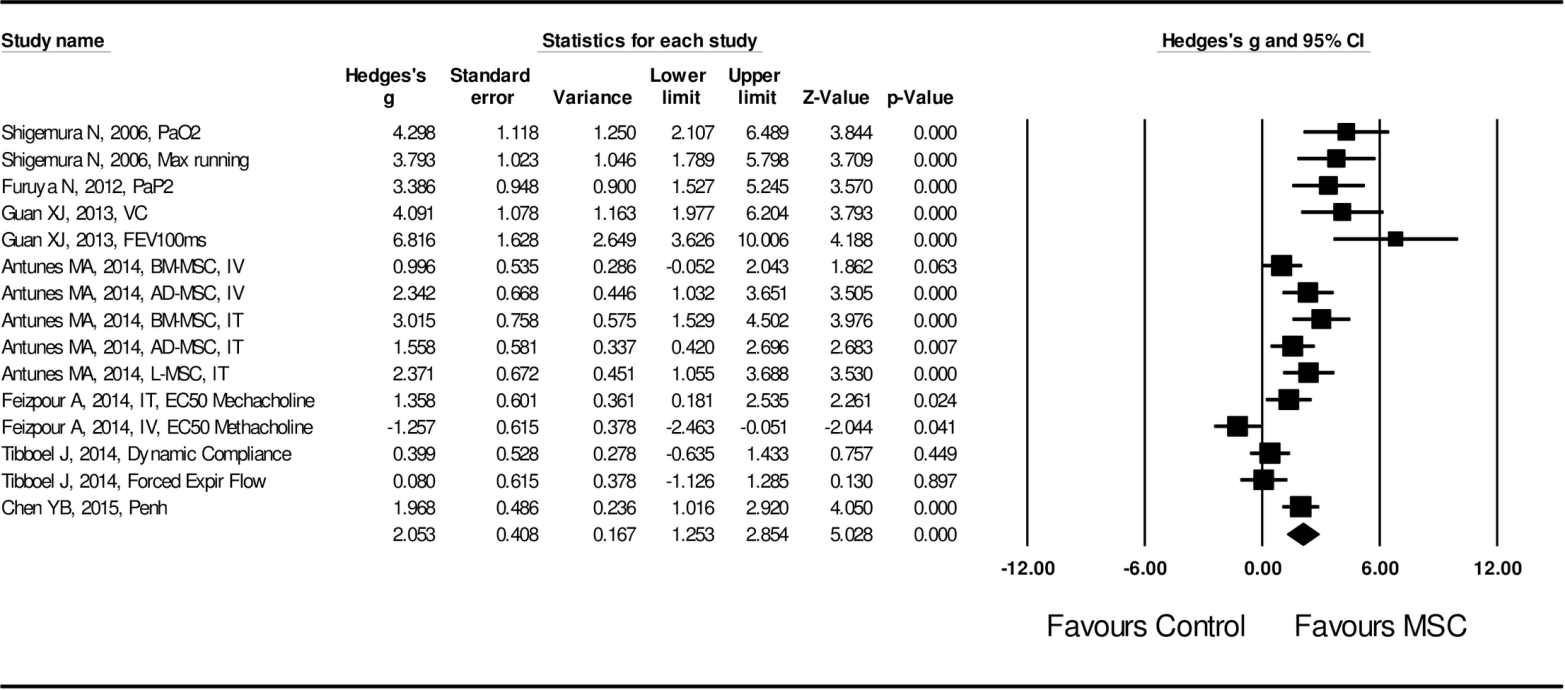
Forest plot for the effect of MSCs on lung function in the COPD models. A random effect model was used due to significant heterogeneity of publications (I^2^ = 80.1, *P* < 0.01). Effect size was assessed by Hedges’s g and 95% CI, and the effect on lung function improvement was in favor of MSC administration (Hedges’s g = 2.053 ± 0.408, 95% CI: 1.253~2.854, *P* < 0.001). Control group was the COPD model without MSC treatment.

Next, effect size of MSCs on pulmonary functions was examined. Specifically, effect on vital capacity (VC), FEV at 100 milliseconds (FEV_100_), dynamic compliance (C_dyn_), mean forced expiratory flow, EC_50_ methacholine (the effective concentration causing 50% of maximum contraction response to a methacholine challenge test), and plethysmograph (Peth) were evaluated. Again, a random effect model was applied in assessing the effect size of MSCs on lung function improvement (I^2^ = 80.1, *P* < 0.01), which was statistically significant in favor of MSC administration (Hedge’s g = 2.053 ± 0.408 with 95% CI: 1.253 ~ 2.854, *P* < 0.001, **Fig 5**).

**Fig 6:**
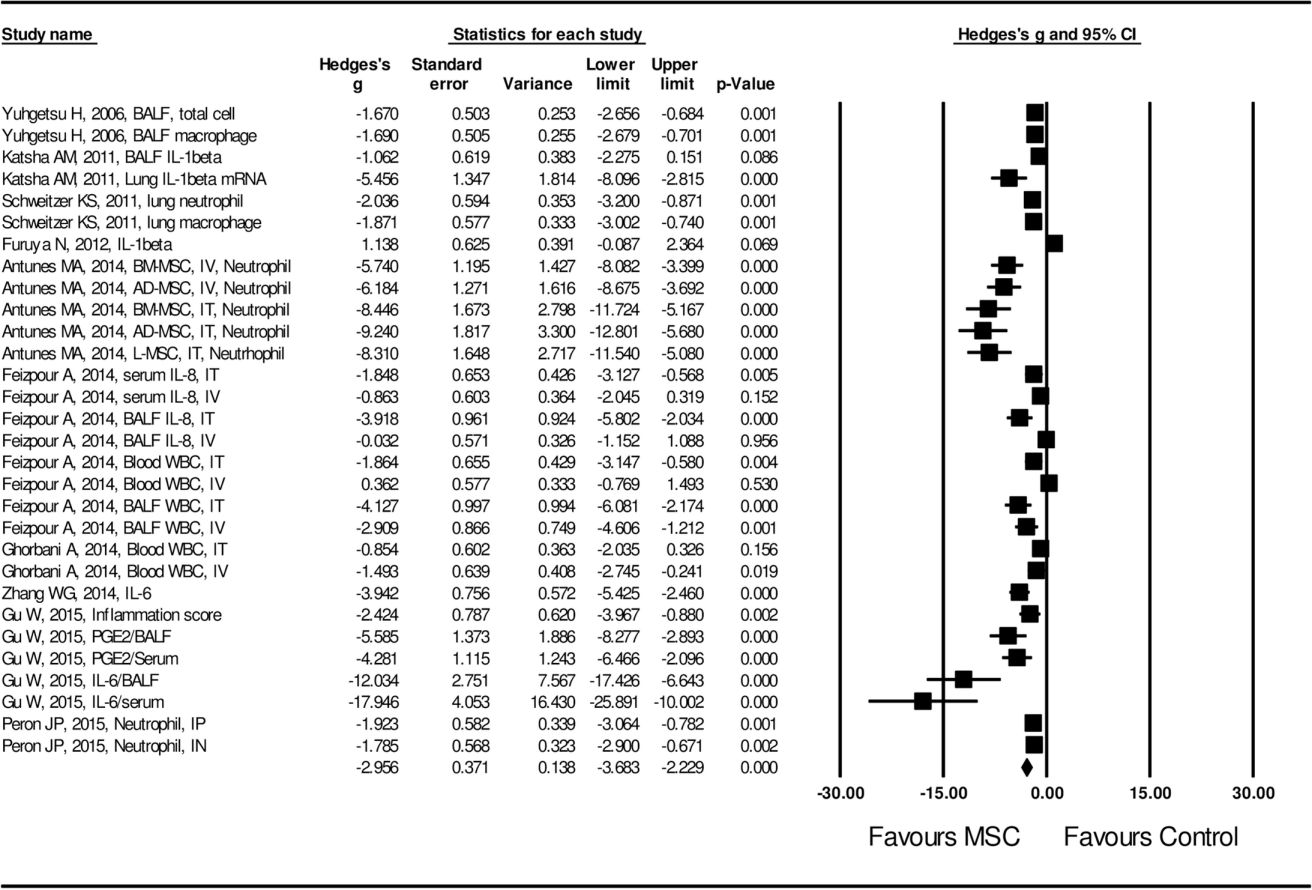
Forest plot for the effect of MSCs on airway infiltration of inflammatory cells or release of pro-inflammatory cytokines in lung or blood. A random effect model was used due to significant heterogeneity of publications (I^2^ = 84.8, *P* < 0.01). Effect size was assessed by Hedges’s g and 95% CI, and the inhibitory effect on airway inflammation and systemic inflammation was in favor of MSC administration (Hedges’s g = -2.956 ± 0.371, 95% CI: -3.683~-2.229, *P* < 0.001). Control group was the COPD model without MSC treatment.

**Fig 7:**
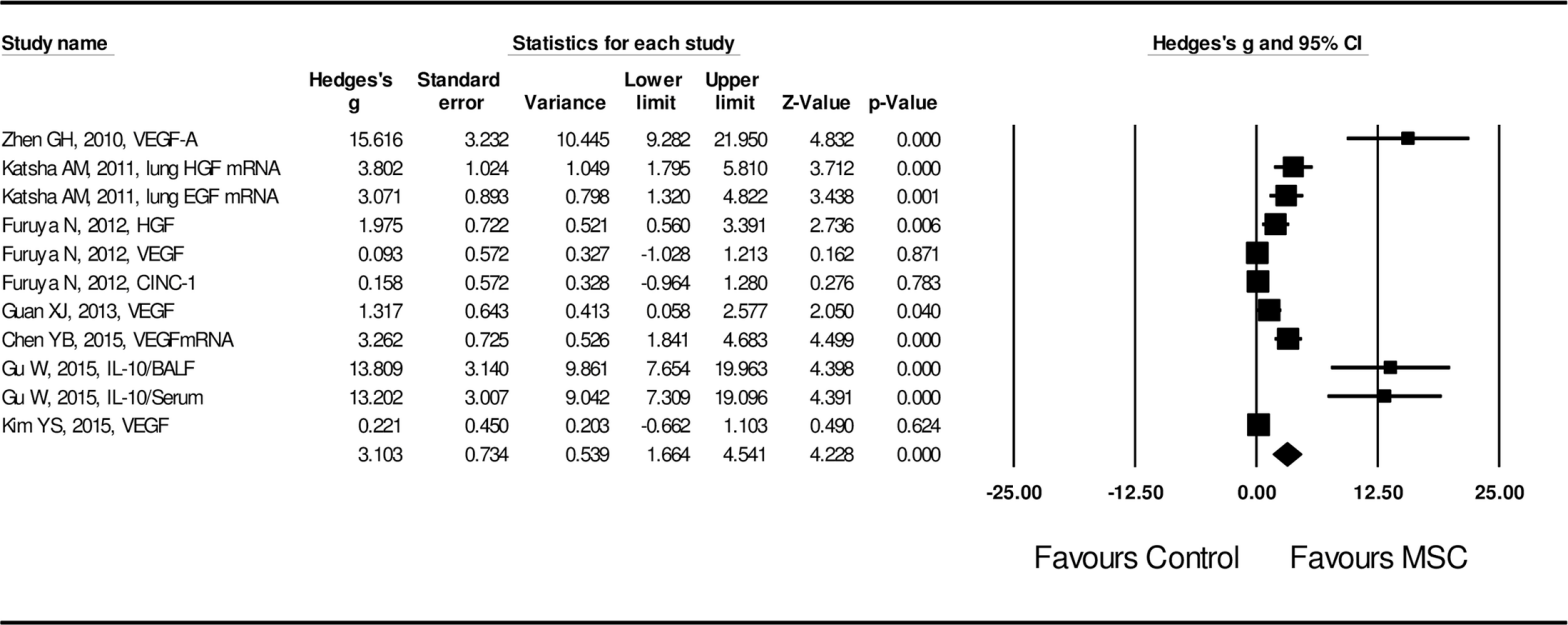
Forest plot for the effect of MSCs on growth factors and anti-inflammatory cytokines. A random effect model was used due to significant heterogeneity of publications (I^2^ = 88.0, *P* < 0.01). Effect size was assessed by Hedges’s g and 95% CI, and the stimulatory effect on growth factors and anti-inflammatory cytokines was in favor of MSC administration (Hedges’s g = 3.103 ± 0.734, 95% CI: 1.664~4.541, *P* < 0.001). Control group was the COPD model without MSC treatment.

Lastly, effect size of MSC administration on inflammation and production of anti-inflammatory cytokines or growth factors stimulating tissue repair was also evaluated. Inflammation was evaluated by the following parameters: infiltration of neutrophils or macrophages, IL-6 release, cyclooxygenase-2 (COX-2) expression, PGE_2_ release, and production of matrix metalloproteinases (MMP-9 and MMP-12) etc. In addition, the effect size of MSCs on the release of IL-10, VEGF, HGF, EGF and TGF-β1 was assessed to evaluate the potential mechanism of MSC on lung tissue repair. MSC administration resulted in inhibition of airway inflammation, and the effect size was statistically significant (Hedge’s g = -2.956 ± 0.371 with 95% CI: -3.683 ~ -2.229, *P* < 0.001, **Fig 6**,) with significant heterogeneity (I^2^ = 84.8, *P* < 0.01). In contrast, MSC administration resulted in up-regulation of anti-inflammatory cytokine (IL-10) and growth factors (VEGF, HGF, EGF and TGF-β). The effect size was also statistically significant (Hedge’s g = 3.103 ± 0.734 with 95% CI: 1.664 ~ 4.541, *P* < 0.001, **Fig 7)** with high heterogeneity (I^2^ = 88.0, *P* < 0.01).

### Publication bias

Publication bias was originally defined as the publication or non-publication of studies depending on the direction and statistical significance of the results [39]. Publication bias was examined by the funnel plot of standard error versus Hedges’s g. As shown in S1–S6 Figs, distribution of the funnel plot was nearly symmetric in MLI, tissue repair parameters, and lung function assay parameters, but it was asymmetric in the remainder of the plots.

## Discussion

Despite recent advances in the treatment of symptoms in COPD patients, the treatment of severe COPD continues to be very challenging and there remains no effective therapy that has been shown to reduce progression of emphysema [40]. Over the past decade, increasing number of preclinical studies have suggested that administration of mesenchymal stem/stromal cells (MSCs) can prevent or have a therapeutic effect in COPD animal models. Based on the preclinical findings, a multi-center clinical trial of MSC administration in the treatment of COPD patients had been conducted, although the results in human studies were less promising compared to the findings of preclinical studies [18]. We systematically reviewed 21 publications of preclinical studies of MSC administration in the treatment of COPD in animal models and further performed a meta-analysis to examine the combined effect of MSC in COPD therapy. The current meta-analysis indicated that MSC administration either by intravenous injection or intra-tracheal instillation resulted in significant reduction of MLI and TUNEL positivity in the animal models of COPD, significant stimulation of lung tissue repair, and significant improvement of lung function. MSCs significantly attenuated airway infiltration of neutrophils and macrophages and production of pro-inflammatory cytokines including IL-1β and IL-6, but significantly stimulated anti-inflammatory cytokine IL-10 and growth factors including VEGF, HGF, EGF, and TGF-β, suggesting MSC administration is an effective approach to treat COPD/emphysema in the animal models and hold promise of future application of MSC administration in COPD patients.

While initial interest of MSC administration in a variety of diseases was centered on the capacity for multi-lineage differentiation of the cells, recently MSCs have been considered potent modulators of disease-associated tissue microenvironments such as milieu of chronic inflammation and autoimmune reaction [41]. Thus, in the past decade, studies on MSCs have been focused on not only direct tissue and organ regeneration but also modulatory effects on damaged and diseased tissues [42]. The anti-inflammatory and immunomodulatory properties of MSCs [6, 7, 43] has been the focus of many of the recently published literature reports. COPD is characterized by chronic airway inflammation and insufficient tissue repair [44]. Therefore, MSC administration could be an effective cellular therapy for COPD[8, 18, 32]. Although there are 8 clinical trials currently ongoing to examine safety and efficacy of MSC administration in COPD patients (Clinicaltrials.gov: NCT02645305, NCT01849159, NCT02348060, NCT02412332, NCT02161744, NCT02041000, NCT02216630, NCT01559051), only two clinical trials of MSC administration in COPD patients have been completed. In this content, Ribeiro-Paes et al from Brazil examined the effects of intravenous infusion of autologous bone marrow mononuclear cells in the treatment of advanced COPD patients (4 cases total) and 12-month follow-up showed a significant improvement in the quality of life as well as a clinically stable condition [45]. The result of a clinical trial conducted by Weiss et al from the USA, however, was disappointing, and found lack of even a trend for efficacy of MSC administration in COPD [18]. Therefore, the current study was designed to systematically review and analyze recent publications of preclinical studies of MSCs and COPD, but not clinical trials.

The preclinical studies provide important evidence of MSC safety, toxicity, therapeutic efficacy and mechanism of MSC action for future human clinical use. In this regard, studies on rodent animal models of COPD have demonstrated that intravenous injection or intra-tracheal instillation of rodent bone marrow MSCs (BM-MSCs) or adipose derived MSCs (AD-MSCs) were safe and effective in attenuating airway injury by ameliorating airway inflammation and apoptosis [8, 28, 38]. In contrast, intravenous injection of mice lung tissue derived MSCs (L-MSCs) resulted in immediate death of the recipient mice, which may be associated with the larger size of the L-MSCs or with cellular clumping resulting in pulmonary embolism [8]. In addition, intra-tracheal instillation of BM-MSC seemed to be superior to intravenous injection in reducing alveolar hyperinflation and collagen fiber content in the elastase-induced emphysema models [8]. These findings suggested that intra-tracheal or intra-bronchial instillation is a preferred and safer way of MSC administration for the treatment of airway diseases.

Studies on pharmacokinetics of MSCs *in vivo* demonstrated that both allogeneic and autologous MSCs appeared to distribute in a similar manner [46]. BM-MSCs distributed mostly in lungs, liver and spleen at early stages (hours) of intravenous injection regardless the injury located in the brain or heart [47, 48]. Consistent with animal model studies, a study on the fate of MSCs examined autopsy materials from 18 patients who had received human leukocyte antigen (HLA)-mismatched MSCs and found that MSC donor DNA was detectable in the lungs, lymph nodes and intestine [49]. Furthermore, no signs of ectopic tissue formation or malignant tumors of MSC-donor origin were found on macroscopic or histological examination [49]. These findings indicate the lung is one of the organs where MSCs initially distribute following systemic administration.

Preclinical models have also provided important opportunities for testing and evaluating the immune response induced by allogeneic MSCs under varying conditions [43]. Allogeneic MSCs are considered to be poorly immunogenic in comparison with other cells and tissues, and thus, human BM-MSCs, AD-MSCs, iPSCs, and cord blood derived MSCs (CBD-MSCs) had been tested and evaluated in the experimental models of emphysema [21–24]. These human MSCs were delivered to rodent recipients either by intravenous injection or by intra-peritoneal injection. Administration of these MSCs significantly reduced airway inflammation, parenchymal lung cell apoptosis and peri-bronchial collagen deposition in the recipient animals of cigarette smoke- or elastase-induced emphysema. These findings suggest that the preclinical studies provide valuable information regarding mechanisms of MSC action, safety, immunogenicity, and *in vivo* kinetics of therapeutically administered MSCs. Moreover, these pre-clinical studies demonstrate that xenogeneic MSC administration is safe and effective, and thus, in addition to autologous MSC, administration of allogeneic human MSC is safe and plausible in clinical trials.

Substantial progress has been made recently in our understanding of the mechanisms of interactions between MSCs and the recipient tissue microenvironment. As a result of such studies MSCs are now known to have anti-inflammatory and immune modulatory effects. In this regard, MSCs are adapted to their microenvironment through either the release of soluble factors such as PGE_2_, kyneurnine, IL-10, TNF-stimulated gene 6 protein (TSG-6), NO, and TGF-β1 [50–55], or context-dependent modification of T helper (Th1/Th2) balance or pro-inflammatory Th17 cell differentiation [55–57]. Consistent with these reports, in the experimental models of COPD, MSCs could also modulate release of inflammation-associated factors, that is, inhibiting pro-inflammatory cytokines or mediators such as IL-1β, TNF-α, IL-6, and PGE_2_ [29, 30, 33, 37], stimulating anti-inflammatory cytokine IL-10 [29], and up-regulating synthesis of growth factors associated with tissue repair such as VEGF, HGF, EGF and TGF-β1 [10, 23, 26, 28, 33, 37].

While the current meta-analysis was carried out on a rigorous systematic review that could avoid publication bias, potential publication bias may exist in the current study [58, 59]. In this regard, funnel plots of MLI, lung tissue repair parameters and lung function assay parameters were nearly symmetrically distributed, suggesting no publication bias might exist in these observations. However, distribution in the funnel plots of TUNEL positivity, inflammation, and growth factors was asymmetric, indicating publication bias may exist in these analyses.

There are several limitations in the current systematic review and meta-analysis. First, studies used different sources of stem cells including bone marrow, adipose tissue, lung tissue, umbilical cord blood, tubal tissue, and amniotic fluid MSCs from human as well as rodents. Second, various routes of MSC administration were used by different investigators, i.e., delivered through intravenous injection, intra-tracheal or intra-nasal instillation, or intra-peritoneal injection. Third, the protocol of assessment of emphysematous lung damage and repair was not standardized in terms of time of MSC delivery and period of observation etc. Due to the aforementioned limitations, publication bias may exist in the current review. Additionally, a random model was used to examine the effect size of MSC therapeutic effects on COPD due to the data heterogeneity. Fourth, all of the animal models of COPD were in acute phase or sub-acute phase of lung tissue injury, which varies greatly from the chronic inflammation-induced lung tissue damage and insufficient repair observed in clinical COPD patients. Fifth, the publication bias was examined only by funnel plot and not further examined by other methods such as Egger’s regression.

Following the completion of the clinical trial by Weiss et al [18], administration of MSCs for COPD treatment is being further tested in eight clinical trials (clinicaltrials.gov: NCT02645305, NCT01849159, NCT02348060, NCT02412332, NCT02161744, NCT02041000, NCT02216630, NCT01559051). These clinical trials were designed to further evaluate efficacy and safety of systemic administration of autologous or allogeneic MSCs in the treatment of COPD. Researchers of the clinical trials anticipate that MSCs will inhibit chronic inflammation in airway, alveoli and endothelium, promote tissue repair through releasing growth factors, and improve patient’s quality of life. We expect that systemic administration of MSCs in COPD patients is safe and will become an effective cellular therapy for COPD in near future.

## Conclusion

Taken together, the recent literature of preclinical studies of MSCs administration in COPD animal models provides a wealth of potentially valuable information regarding *in vivo* safety, immunogenicity, pharmacokinetics, and mechanisms of action of therapeutically administered MSCs. These preclinical studies demonstrated that intravenous injection or intra-tracheal delivery of MSCs (regardless BM-MSC, AD-MSC, or CBD-MSC) is safe and effective in the therapy of COPD experimental models. The current systematic review and meta-analysis suggest a promising role for MSC administration in COPD treatment. The mechanisms of MSCs in pre-clinical COPD treatment may be associated with attenuating airway inflammation as well as stimulating lung tissue repair.

## Supporting Information

S1 Checklist PRISMA 2009 Checklist. (DOC)

S1 Fig Funnel plot of MLI. (TIFF)

S2 Fig Funnel plot of TUNEL. (TIFF)

S3 Fig Funnel plot of tissue repair parameters. (TIFF)

S4 Fig Funnel plot of lung function. (TIFF)

S5 Fig Funnel plot of inflammation (TIFF)

S6 Fig Funnel plot of growth factors. (TIFF)
