# Supplementary figures and images for: Preclinical Studies of Mesenchymal Stem Cell (MSC) Administration in Chronic Obstructive Pulmonary Disease (COPD): A Systematic Review and Meta-Analysis
Source: PLoS One. 2016 Jun 9;11(6):e0157099. doi: 10.1371/journal.pone.0157099 (PMC4900582; doi:10.1371/journal.pone.0157099)

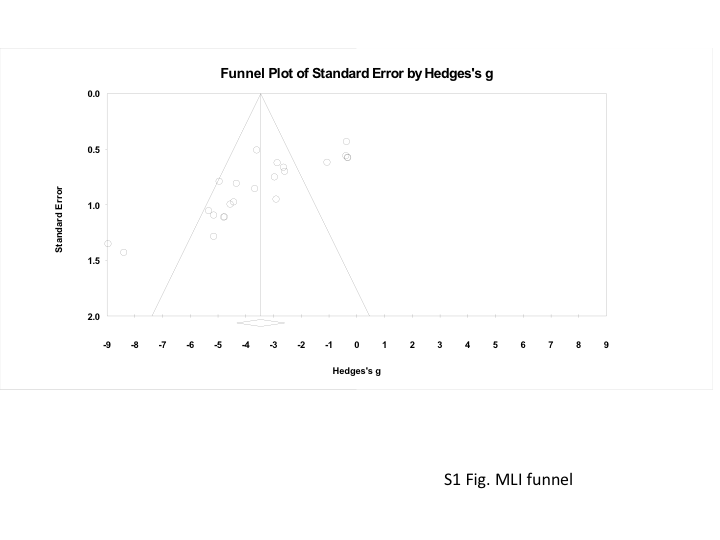

Supplement: S1 Fig — (TIFF) [file pone.0157099.s002.tiff]

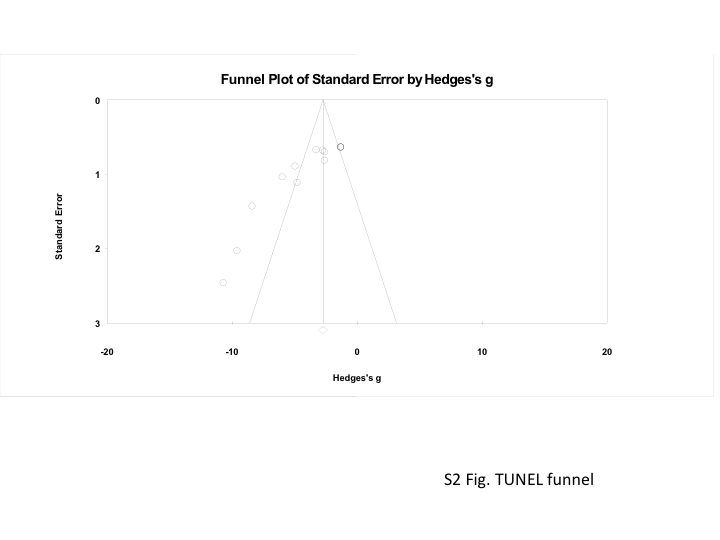

Supplement: S2 Fig — (TIFF) [file pone.0157099.s003.tiff]

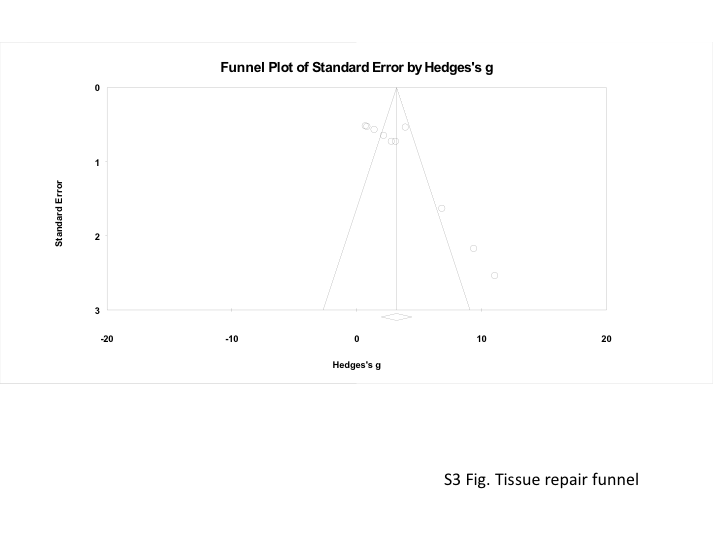

Supplement: S3 Fig — (TIFF) [file pone.0157099.s004.tiff]

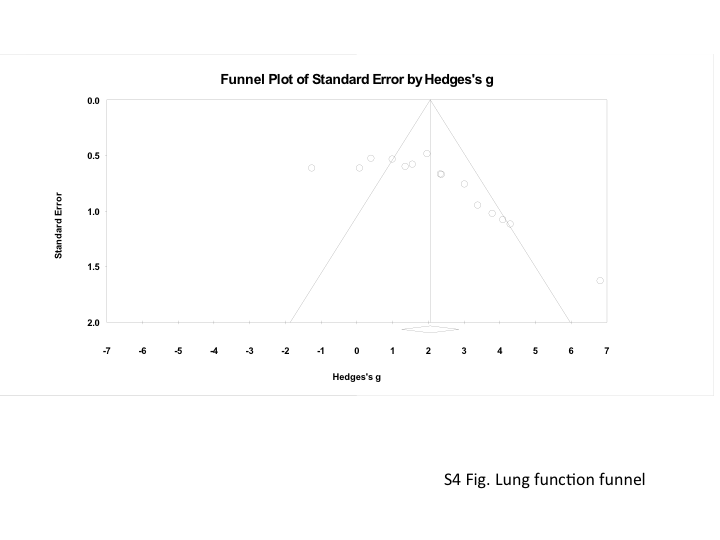

Supplement: S4 Fig — (TIFF) [file pone.0157099.s005.tiff]

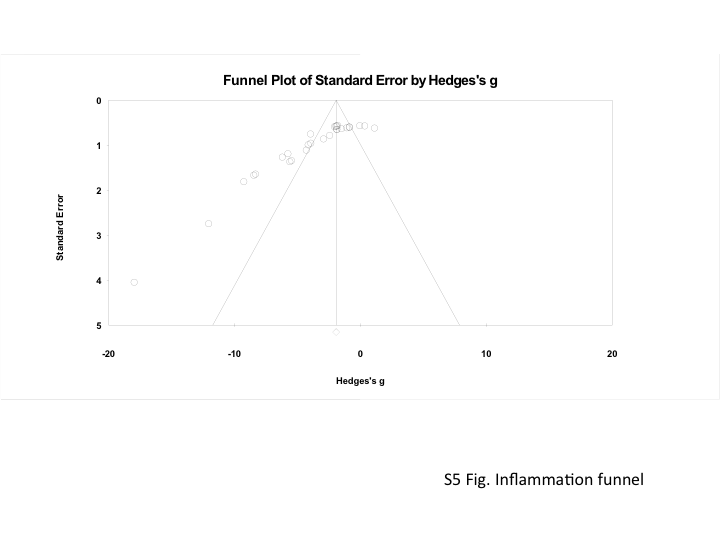

Supplement: S5 Fig — (TIFF) [file pone.0157099.s006.tiff]

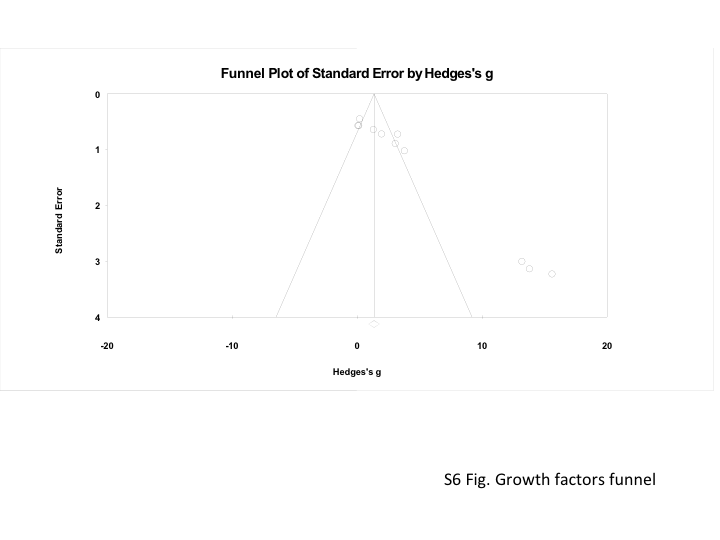

Supplement: S6 Fig — (TIFF) [file pone.0157099.s007.tiff]
